# Abnormally Low and High Ankle-Brachial Indices Are Independently Associated with Increased Left Ventricular Mass Index in Chronic Kidney Disease

**DOI:** 10.1371/journal.pone.0044732

**Published:** 2012-09-05

**Authors:** Ho-Ming Su, Tsung-Hsien Lin, Po-Chao Hsu, Chun-Yuan Chu, Wen-Hsien Lee, Szu-Chia Chen, Chee-Siong Lee, Wen-Chol Voon, Wen-Ter Lai, Sheng-Hsiung Sheu

**Affiliations:** 1 Division of Cardiology, Department of Internal Medicine, Kaohsiung Medical University Hospital, Kaohsiung Medical University, Kaohsiung, Taiwan; 2 Division of Nephrology, Department of Internal Medicine, Kaohsiung Medical University Hospital, Kaohsiung Medical University, Kaohsiung, Taiwan; 3 Department of Internal Medicine, Kaohsiung Municipal Hsiao-Kang Hospital, Kaohsiung Medical University, Kaohsiung, Taiwan; 4 Faculty of Mecicine, College of Medicine, Kaohsiung Medical University, Kaohsiung, Taiwan; Baker IDI Heart and Diabetes Institute, Australia

## Abstract

Abnormally low and high ankle-brachial indices (ABIs) are associated with high cardiovascular morbidity and mortality in patients with chronic kidney disease (CKD), but the mechanisms responsible for the association are not fully known. This study is designed to assess whether there is a significant correlation between abnormal ABI and echocariographic parameters in patients with CKD stages 3–5. A total of 684 pre-dialysis CKD patients were included in the study. The ABI was measured using an ABI-form device. Patients were classified into ABI <0.9, ≥0.9 to <1.3, and ≥1.3. Clinical and echocariographic parameters were compared and analyzed. Compared with patients with ABI of ≥0.9 to <1.3, the values of left ventricular mass index (LVMI) were higher in patients with ABI <0.9 and ABI ≥1.3 (*P*≤0.004). After the multivariate analysis, patients with ABI <0.9 (β = 0.099, *P* = 0.004) and ABI ≥1.3 (β = 0.143, *P*<0.001) were independently associated with increased LVMI. Besides, increased LVMI (odds ratio, 1.017; 95% confidence interval, 1.002 to 1.033; *P* = 0.031) was also significantly associated with ABI <0.9 or ABI ≥1.3. Our study in patients of CKD stages 3–5 demonstrated abnormally low and high ABIs were positively associated with LVMI. Future studies are required to determine whether increased LVMI is a causal intermediary between abnormal ABI and adverse cardiovascular outcomes in CKD.

## Introduction

The ankle-brachial index (ABI) is reported to be a good marker for atherosclerosis and an ABI <0.9 is useful in the diagnosis of peripheral artery occlusive disease (PAOD) [1], [2]. In addition, an ABI ≥1.3 is considered to indicate medial artery calcification (MAC) [3]. High prevalence of PAOD and increased MAC are frequently noted in patients with chronic kidney disease (CKD) [4], [5], which may through some processes including vascular calcification, inflammatory and coagulation pathways alterations, oxidative stress, malnutrition and infection [6]–[8]. Moreover, either abnormally low or high ABIs can predict overall and cardiovascular mortality in patients with chronic renal failure [9]–[11]. However, the mechanisms responsible for the association are not fully known.

Recent studies in hypertensive patients and in general population reported abnormally low and high ABIs were independently associated with increased left ventricular mass [12], [13]. Abnormally low and high ABIs are affected by or linked to various risk factors for left ventricular hypertrophy (LVH), such as arterial stiffness, hypertension and coexisting atherosclerosis [14], [15]. LVH is frequently encountered in patients with CKD because of pressure and volume overload, increased arterial stiffness, anemia, inflammatory status, protein malnutrition and sodium/water retention, which were also key causes for atherosclerosis in this population [16]–[19]. LVH and peripheral artery disease are common cardiovascular complications in CKD patients, in whom there may be a close and cause-effect relationship. However, there are limited studies to evaluate the association between abnormal ABI and left ventricular mass index (LVMI) in CKD patients, whom frequently have many atherosclerotic risk factors and increased left ventricular mass. Accordingly, the aim of this study is to assess whether there is an independent association between abnormal ABI and increased LVMI in patients with CKD stages 3–5.

## Subjects and Methods

### Study Patients and Design

The study was conducted in a regional hospital in southern Taiwan. We consecutively enrolled patients with CKD of stages 3 to 5 [20] from our Outpatient Department of Internal Medicine from July 2009 to February 2010. Patients with atrial fibrillation, inadequate image visualization and ABI <0.9 in one leg and ABI ≥1.3 in the other leg were excluded. A total of 684 patients (mean age 65.5±12.5 years, 401 males/283 females) were included.

### Ethics Statement

The study protocol was approved by the institutional review board of the Kaohsiung Medical University Hospital (KMUH-IRB-20120058). Informed consents have been obtained in written form from patients and all clinical investigation was conducted according to the principles expressed in the Declaration of Helsinki. The patients gave consent for the publication of the clinical details.

**Table 1:** Comparison of clinical and echocardiographic characteristics between patients with normal and abnormal ABI.

Characteristics	ABI ≥0.9 to <1.3 (n = 556)	ABI <0.9 (n = 62)	ABI ≥1.3 (n = 66)
Age (year)	64.6±12.4	75.3±9.6^**^	63.6±12.1
Male gender (%)	57.7	61.3	63.6
Smoking history (%)	33.7	46.7	31.8
Diabetes mellitus (%)	35.3	61.3^**^	54.5*
Hypertension (%)	76.3	90.3*	84.8
Coronary artery disease (%)	15.2	35.5^**^	21.2
Cerebrovascular disease (%)	8.8	28.1^**^	8.2
Stage of CKD			
Stage 3 (%)	76.1	59.7*	59.1*
Stage 4 (%)	12.6	22.6	13.6
Stage 5 (%)	11.3	17.7	27.3
Systolic blood pressure (mmHg)	140.7±22.4	144.1±21.5	138.6±19.3
Diastolic blood pressure (mmHg)	79.2±12.8	73.9±11.7*	76.8±12.6
Pulse pressure (mmHg)	61.6±15.1	70.2±17.3^**^	61.8±12.5
Body mass index (kg/m2)	25.7±4.0	24.3±3.3	26.9±5.1
Laboratory parameters			
Albumin (g/dL)	4.12±0.40	3.90±0.50*	4.08±0.40
Fasting glucose (mg/dL)	113.6±39.4	135.2±67.4*	127.3±53.7
Triglyceride (mg/dL)	126 (88–187)	127 (86–175)	117 (87.75–175.25)
Total cholesterol (mg/dL)	189.5±40.2	192.2±47.1	186.4±37.0
Hematocrit (%)	38.5±6.0	35.2±6.2^**^	36.0±6.5*
eGFR (mL/min/1.73 m2)	40.8±16.1	33.9±15.4*	33.3±18.6*
Uric acid (mg/dL)	7.4±2.1	7.8±2.6	7.7±2.2
Proteinuria (%)	53.5	71.4	77.3*
Medications			
Aspirin use (%)	18.3	20.0	21.4
ACEI and/or ARB use (%)	61.9	75.4*	53.4
Non-ACEI/ARB antihypertensive drug use (%)	72.1	83.9*	77.3
Statin use (%)	21.9	25.0	18.9
Echocardiographic data			
LVMI (g/m^2^)	129.6±45.7	150.3±58.1*	154.7±50.3^**^
LVH (%)	65.3	74.2	83.3*
LVEF (%)	62.5±13.7	58.7±16.7	62.0±13.5
E-wave deceleration time (ms)	211.9±67.6	215.9±85.5	225.3 ±72.5
E/A	0.95±0.54	0.99±0.46	0.95±0.49

Abbreviations. ABI, ankle-brachial index; CKD, chronic kidney disease; eGFR, estimated glomerular filtration rate; ACEI, angiotensin converting enzyme inhibitor; ARB, angiotensin II receptor blocker; LVMI, left ventricular mass index; LVH, left ventricular hypertrophy; LVEF, left ventricular ejection fraction; E, peak early transmitral filling wave velocity; A, peak late transmitral filling wave velocity.

* *P*<0.05, ^**^ *P*<0.001 compared with ABI of ≥0.9 to<1.3 in both legs.

**Table 2:** Comparison of clinical and echocardiographic characteristics between patients without and with LVH.

Characteristics	Non-LVH (n = 302)	LVH (n = 382)
ABI		
≥0.9 to<1.3	87.7	78.2*
<0.9 in either leg	7.3	9.9
≥1.3 in either leg	5.0	11.9*
Age (year)	63.8±12.2	66.3±12.6*
Male gender (%)	69.1	53.7^**^
Smoking history (%)	37.5	30.5
Diabetes mellitus (%)	39.1	39.7
Hypertension (%)	75.9	79.5
Coronary artery disease (%)	17.4	17.8
Cerebrovascular disease (%)	12.1	9.7
Stage of CKD		
Stage 3 (%)	74.1	72.4
Stage 4 (%)	16.8	12.1
Stage 5 (%)	9.1	15.5*
Systolic blood pressure (mmHg)	136.2±19.7	143.0±22.7^**^
Diastolic blood pressure (mmHg)	78.1±11.5	78.6±13.3
Pulse pressure (mmHg)	58.1±13.7	64.4±15.6^**^
Body mass index (kg/m2)	25.0±3.8	26.0±4.2*
Laboratory parameters		
Albumin (g/dL)	4.19±0.34	4.03±0.45^**^
Fasting glucose (mg/dL)	120.0±50.3	115.2±41.1
Triglyceride (mg/dL)	118.5 (88–189.5)	132 (88–182)
Total cholesterol (mg/dL)	188.5±38.7	189.9±41.4
Hematocrit (%)	39.1±5.9	37.4±6.2*
eGFR (mL/min/1.73 m2)	41.4±16.0	38.6±16.8*
Uric acid (mg/dL)	7.4±2.1	7.5±2.1
Proteinuria (%)	50.8	64.6*
Medications		
Aspirin use (%)	17.0	21.0
ACEI and/or ARB use (%)	62.8	62.2
Non-ACEI/ARB antihypertensive drug use (%)	66.8	76.9*
Statin use (%)	20.8	22.4
Echocardiographic data		
LVMI (g/m^2^)	89.2±18.2	155.1±43.1^**^
LVEF (%)	65.8±11.6	60.4±14.7^**^
E-wave deceleration time (ms)	214.7±64.4	213.0±72.3
E/A	0.95±0.57	0.96±0.51

* *P*<0.05, ^**^ *P*<0.001 compared with patients without LVH. Abbreviations are the same as in Table 1.

### Evaluation of cardiac structure and function

The echocardiographic examination was performed by one experienced cardiologist with a VIVID 7 (General Electric Medical Systems, Horten, Norway), with the participant respiring quietly in the left decubitus position. The cardiologist was blind to the other data. Two-dimensional and two-dimensionally guided M-mode images were recorded from the standardized views. The echocardiographic measurements included left ventricular internal diameter in diastole (LVIDd), left ventricular posterior wall thickness in diastole (LVPWTd), interventricular septal wall thickness in diastole (IVSTd), E-wave deceleration time, peak early transmitral filling wave velocity (E) and peak late transmitral filling wave velocity (A). Left ventricular systolic function was assessed by left ventricular ejection fraction (LVEF). Left ventricular mass was calculated using Devereux-modified method, i.e. left ventricular mass  = 1.04×[(IVSTd + LVIDd + LVPWTd)^3^ – LVIDd^3^] – 13.6g [21]. LVMI was calculated by dividing left ventricular mass by body surface area. LVH was defined as suggested by the American Society of Echocardiography/European Society of Echocardiography chamber quantification guidelines [22]. Left ventricular relative wall thickness (LVRWT) was calculated as the ratio of 2×LVPWTd / LVIDd. Cardiac remodeling was defined as LVRWT more than 0.42 without LVH. Concentric LVH was defined as LVMI more than 115 g/m^2^ in men and more than 95 g/m^2^ in women, with LVRWT more than 0.42; eccentric LVH was defined as LVMI more than 115 g/m^2^ in men and more than 95 g/m^2^ in women, with LVRWT less than 0.42. In addition, thirty patients were randomly selected for evaluation of the interobserver variability of left ventricular mass measurement by 2 independent observers. Mean percent error was calculated as the absolute difference divided by the average of the two observations. The interobserver mean percent error for left ventricular mass measurement in this study was 9.8±5.8%.

**Table 3:** ABI category in different left ventricular geometry.

Characteristics	Normal (n = 244)	Concentric remodeling (n = 58)	Eccentric LVH (n = 284)	Concentric LVH (n = 98)	*P*
ABI					
≥0.9 to<1.3 (%)	87.7	87.7	80.4*	74.2*	0.001
<0.9 in either leg (%)	5.8	10.8	9.6	10.4	0.041
≥1.3 in either leg (%)	6.5	1.5	10.0	15.3	0.018

* *P*<0.05, ^**^ *P*<0.001 compared with patients with normal left ventricular geometry. Abbreviations are the same as in Table 1.

### Assessment of ABI

The values of ABI were measured by using an ABI-form device (VP1000; Colin Co. Ltd., Komaki, Japan), which automatically and simultaneously measured blood pressures in both arms and ankles using an oscillometric method [23]–[25]. The ABI was calculated by the ratio of the ankle systolic blood pressure divided by the higher systolic blood pressure of the arms. The ABI measurement was done once in each patient. Patients were classified into ABI <0.9 in either leg, ≥0.9 to <1.3 in both legs and ≥1.3 in either leg.

### Collection of demographic, medical, and laboratory data

Demographic and medical data including age, gender, smoking history (ever *versus* never) and comorbid conditions were obtained from medical records or interviews with patients. The body mass index (BMI) was calculated as the ratio of weight in kilograms divided by square of height in meters. Laboratory data were measured from fasting blood samples using an autoanalyzer (Roche Diagnostics GmbH, D-68298 Mannheim COBAS Integra 400). Serum creatinine was measured by the compensated Jaffé (kinetic alkaline picrate) method in a Roche/Integra 400 Analyzer (Roche Diagnostics, Mannheim, Germany) using a calibrator traceable to isotope-dilution mass spectrometry [26]. The value of estimated glomerular filtration rate (eGFR) was calculated using the 4-variable equation in the Modification of Diet in Renal Disease (MDRD) study [27]. Proteinuria was examined by dipsticks (Hema-Combistix, Bayer Diagnostics). A test result of 1+ or more was defined as positive. Blood and urine samples were obtained within 1 month of enrollment. In addition, information regarding patient medications including aspirin, angiotensin converting enzyme inhibitors (ACEIs), angiotensin II receptor blockers (ARBs), non ACEI/ARB antihypertensive drugs and HMG-CoA reductase inhibitors (statins) during the study period was obtained from medical records.

### Statistical analysis

Statistical analysis was performed using SPSS 15.0 for windows (SPSS Inc. Chicago, USA). Data are expressed as percentages, mean ± standard deviation or median (25^th^–75^th^ percentile) for triglyceride. The differences between groups were checked by Chi-square test for categorical variables or by independent t-test for continuous variables. The relationship between two continuous variables was assessed by a bivariate correlation method (Pearson's correlation). Multiple linear and logistic regression analyses were used to identify the factors associated with LVMI and abnormal ABI, respectively. Significant variables in univariate analysis were selected for multivariate analysis. A difference was considered significant if the *P* value was less than 0.05.

## Results

The mean age of the 684 patients was 65.5±12.5 years. The prevalences of ABI <0.9 and ≥1.3 were 9.1% and 9.6%, respectively. The differences between patients with normal and abnormal ABI were shown in Table 1. Compared with patients with normal ABI, patients with ABI <0.9 were found to have an older age, higher prevalence of diabetes mellitus (DM), hypertension, coronary artery disease and cerebrovascular disease, lower prevalence of stage 3 CKD, lower diastolic blood pressure, higher pulse pressure, lower BMI, lower serum albumin, lower fasting glucose, lower hematocrit, lower eGFR, higher percentage of ACEI and/or ARB and non-ACEI/ARB antihypertensive drug use and higher LVMI (*P* = 0.009). In addition, compared with patients with normal ABI, patients with ABI ≥1.3 were found to have higher prevalence of DM, lower prevalence of stage 3 CKD, lower hematocrit, lower eGFR, higher LVMI (*P*<0.001) and higher prevalence of LVH (*P* = 0.009). The LVMI values in ABI ≥0.9 to <1.3, <0.9 and ≥1.3 were 129.6±45.7, 150.3±58.1 and 154.7±50.3 g/m^2^, respectively. Additionally, the prevalences of LVH in these patients were 65.3%, 74.2% and 83.3%, respectively.

The comparison of clinical and echocardiographic characteristics between patients with and without LVH was shown in Table 2. Compared with patients without LVH, patients with LVH had lower prevalence of normal ABI, higher prevalence of ABI ≥1.3, an older age, a lower percentage of male patients, higher prevalence of stage 5 CKD, higher systolic blood pressure, wider pulse pressure, higher BMI, lower albumin, lower hematocrit, lower eGFR, higher prevalence of proteinuria, higher percentage of having received non-ACEI and/or ARB therapy, higher LVMI and lower LVEF. In addition, Table 3 showed the percentage of ABI category in different left ventricular geometry.

Table 4 showed the determinants of LVMI in our study patients. The LVMI correlated positively with ABI <0.9 and ≥1.3 (*versus* ABI ≥0.9 to <1.3), systolic blood pressure, pulse pressure, BMI, non-ACEI/ARB antihypertensive drug use and E/A, but negatively with serum albumin level, total cholesterol, hematocrit, eGFR and LVEF. Further forward multivariate analysis showed that increased LVMI was correlated with ABI <0.9 (β = 0.099, *P* = 0.004), ABI ≥1.3 (β = 0.143, *P*<0.001), increased systolic blood pressure, increased BMI and decreased LVEF.

**Table 4:** Determinants of LVMI in study patients.

Parameter	Univariate		Multivariate (Forward)	
	Standardized coefficient β	*P*	Standardized coefficient β	*P*
ABI (*versus* ≥0.9 to <1.3)	Reference		Reference	
<0.9 in either leg	0.123	0.001	0.099	0.004
≥1.3 in either leg	0.154	<0.001	0.143	<0.001
Age (per 1 year)	0.062	0.103	-	-
Male *versus* female	0.058	0.130	-	-
Smoking (ever *versus* never)	0.077	0.244		
Diabetes mellitus	0.028	0.467	-	-
Hypertension	0.044	0.249	-	-
Coronary artery disease	0.001	0.987	-	-
Cerebrovascular disease	0.016	0.684	-	-
Systolic blood pressure (per 1 mmHg)	0.165	<0.001	0.201	<0.001
Diastolic blood pressure (per 1 mmHg)	0.074	0.053	-	-
Pulse pressure (per 1 mmHg)	0.176	<0.001	-	-
Body mass index (per 1 kg/m2)	0.097	0.011	0.137	<0.001
Laboratory parameters				
Albumin (per 1 g/dL)	−0.294	<0.001	-	-
Fasting glucose (per 1 mg/dL)	−0.060	0.166	-	-
Triglyceride (log per 1 mg/dL)	−0.056	0.175	-	-
Total cholesterol (per 1 mg/dL)	−0.090	0.029	-	-
Hematocrit (per 1%)	−0.119	0.003	-	-
eGFR (per 1 mL/min/1.73 m2)	−0.140	<0.001	-	-
Uric acid (per 1 mg/dL)	0.033	0.446	-	-
Proteinuria	0.174	0.010	-	-
Medications				
Aspirin use	0.147	0.073	-	-
ACEI and/or ARB use	0.017	0.681	-	-
Non-ACEI/ARB antihypertensive drug use	0.088	0.022	-	-
Statin use	−0.029	0.497	-	-
Echocardiographic data				
LVEF (per 1%)	−0.358	<0.001	−0.375	<0.001
E-wave deceleration time (per 1 ms)	−0.037	0.337	-	-
E/A (per 1)	0.079	0.040	-	-

Values expressed as standardized coefficient β. Abbreviations are the same as in Table 1.

Table 5 shows the determinants of abnormal ABI (ABI <0.9 or ≥1.3) in study patients. In the univariate analysis, abnormal ABI was found to be significantly associated with increased age, a history of DM, hypertension, coronary artery disease, and cerebrovascular disease, decreased diastolic blood pressure, increased pulse pressure, decreased albumin, increased fasting glucose, decreased hematocrit, decreased eGFR, proteinuria and increased LVMI. In the forward multivariate analysis, a history of coronary artery disease, increased fasting glucose, decreased hematocrit and increased LVMI (odds ratio [OR], 1.017; 95% confidence interval [CI], 1.002 to 1.033; *P* = 0.031) were independent risk factors for abnormal ABI.

**Table 5:** Determinants of abnormal ABI (ABI <0.9 or ≥1.3) in study patients.

Parameter	Univariate		Multivariate (Forward)	
	OR (95% CI)	*P*	OR (95% CI)	*P*
Age (per 1 year)	1.033 (1.016–1.050)	<0.001	-	-
Male *versus* female	1.220 (0.822–1.812)	0.324	-	-
Smoking (ever *versus* never)	1.199 (0.579–2.482)	0.625	-	-
Diabetes mellitus	2.517 (1.701–3.723)	<0.001	-	-
Hypertension	2.179 (1.246–3.812)	0.006	-	-
Coronary artery disease	2.175 (1.387–3.412)	0.001	5.854 (1.635–20.953)	0.007
Cerebrovascular disease	2.232 (1.272–3.917)	0.005	-	-
Systolic blood pressure (per 1 mmHg)	1.001 (0.992–1.010)	0.795	-	-
Diastolic blood pressure (per 1 mmHg)	0.975 (0.960–0.991)	0.003	-	-
Pulse pressure (per 1 mmHg)	1.018 (1.006–1.031)	0.004	-	-
Body mass index (per 1 kg/m2)	0.996 (0.950–1.045)	0.877	-	-
Laboratory parameters				
Albumin (per 1 g/dL)	0.527 (0.310–0.896)	0.018	-	-
Fasting glucose (per 1 mg/dL)	1.007 (1.003–1.012)	0.001	1.010 (1.001–1.019)	0.026
Triglyceride (log per 1 mg/dL)	0.782 (0.320–1.909)	0.589	-	-
Total cholesterol (per 1 mg/dL)	1.000 (0.995–1.005)	0.919	-	-
Hematocrit (per 1%)	0.926 (0.895–0.957)	<0.001	0.916 (0.855–0.980)	0.011
eGFR (per 1 mL/min/1.73 m2)	0.975 (0.964–0.986)	<0.001	-	-
Uric acid (per 1 mg/dL)	1.078 (0.976–1.191)	0.139	-	-
Proteinuria	2.606 (1.162–5.845)	0.020	-	-
Medications				
Aspirin use	1.178 (0.399–3.484)	0.766	-	-
ACEI and/or ARB use	1.109 (0.727–1.694)	0.631	-	-
Non-ACEI/ARB antihypertensive drug use	1.593 (0.991–2.560)	0.055	-	-
Statin use	1.002 (0.599–1.677)	0.993	-	-
Echocardiographic data				
LVMI (per 1 g/m^2^)	1.009 (1.005–1.013)	<0.001	1.017 (1.002–1.033)	0.031
LVEF (per 1%)	0.990 (0.977–1.003)	0.126	-	-
E-wave deceleration time (per 1 ms)	1.002 (0.999–1.005)	0.196	-	-
E/A (per 1)	1.045 (0.730–1.496)	0.810	-	-

Values expressed as odds ratio (OR) and 95% confidence interval (CI). Abbreviations are the same as in Table 1.

There were several risk factors which affected ABI, LVMI, and CKD status like DM and hypertension. We performed another analysis to examine the inter-relationship between these factors. The interaction between DM and abnormal ABI on LVH was statistically significant (OR, 2.410; 95% CI, 1.294 to 4.488; *P* = 0.006). Similarly, the interaction between hypertension and abnormal ABI on LVH was also statistically significant (OR, 2.033; 95% CI, 1.246 to 3.318; *P* = 0.005).

## Discussion

Previous studies reported a U-shaped relationship between ABI values and cardiovascular events in various populations [10], [28], [29]. However, the mechanisms responsible for the association are not fully known. In the present study, we evaluated the association of abnormal ABI with LVMI in CKD stage 3–5 patients. We found that either abnormally low or high ABI was independently associated with increased LVMI.

The ABI is a simple, non-invasive and reliable diagnostic tool for peripheral artery disease. An ABI <0.9 have not only been established as a reliable diagnostic marker for PAOD with high sensitivity and specificity, but also a strong predictor for overall and cardiovascular mortality in patients with chronic renal failure [9]–[11]. PAOD and LVH are highly prevalent in CKD patients [5], [30]. Many factors including pressure overload, renal anemia, malnutrition and inflammatory status were not only main causes for development of LVH, but also for atherosclerosis and increased arterial stiffness in patients with CKD [30], [31]. Previous studies demonstrated that the ABI value in the LVH group was significantly lower than that in the non-LVH group and was independently and reversely associated with LVMI [32], [33]. These results suggested that low ABI might be related to LVH. Atherosclerosis directly caused a decrease in blood perfusion in the lower extremities and an increase in arterial wall stiffness, contributing to decreasing ABI and arterial distensibility, and then final progressed to LVH [5], [34], [35]. Reversely, LVH caused left ventricular systolic and diastolic dysfunction and a decrease in cardiac output, which further deteriorated blood perfusion of the extremities and promoted the progression of peripheral arterial disease and decrease of ABI. Our study also consistently revealed there was a higher LVH prevalence and higher LVMI value in patients with ABI <0.9 and low ABI was independently associated with increased LVMI in CKD patients.

Falsely elevated pressures or incompressible arteries at ankle level are common among patients with extensive vascular calcification of the lower extremities, which may occur in those with diabetes or renal insufficiency [3], [5]. The abnormally high ABI value or incompressible arteries had been interpreted as the presence of MAC [3]. Patients having abnormally high ABI also had poor prognosis for all-cause and cardiovascular mortality in general population and also in chronic renal failure patients [10], [11], [28], [29]. In rodent studies, experimentally induced MAC resulted in greater arterial stiffness and left ventricular mass due to the amount of vascular calcium content. The correlation between MAC and left ventricular mass persistently existed independently of presence or absence of change in mean arterial pressure or arterial diameter, suggesting that MAC might lead to greater left ventricular mass through nonatherosclerotic pathways [15]. Consistent with the experimental model, Ix JH et al. [13] evaluated the association between high ABI and LVMI in 4972 MESA (multi-Ethic Study of atherosclerosis) participants without clinical cardiovascular disease. They found either abnormally low or high ABI was significantly associated with greater LVMI. Besides, they also found that the association between high ABI and LVMI is not attenuated with adjustment of markers of subclinical atherosclerosis, such as common and internal carotid intima media thickness and natural log-transformed coronary artery calcification [13]. Our study showed there was a higher LVH prevalence and higher LVMI value in patients with ABI ≥1.3 and high ABI was independently associated with increased LVMI. Hence, higher left ventricular mass might play a role between high ABI and poor cardiovascular prognosis.

Cardiovascular disease is the leading cause of morbidity and mortality in patients with CKD, presumably due to accumulation of risk factors for LVH and atherosclerosis [36], [37]. Previous studies demonstrated that diabetes, renal anemia, inflammatory status, protein malnutrition and hypertension played roles in the development of LVH and peripheral artery disease in CKD patients [4], [10], [19]. In our study, compared with normal ABI, abnormally low and high ABIs had several factors of increased risk for LVH, such as higher prevalence of DM, hypertension, coronary artery disease and cerebrovascular disease, more advanced CKD stage, higher pulse pressure, lower serum albumin and lower hematocrit. Even after adjustment for these factors, abnormally low and high ABIs were still independently associated with higher LVMI.

Our results demonstrated that abnormally low and high ABIs were significantly correlated with increased LVMI in patients with CKD stages 3–5. Future studies are required to determine whether increased LVMI is a causal intermediary between abnormal ABI and adverse cardiovascular outcomes in CKD.
